# Handcrafting Joy to Support Both the Patient and Clinician Experience

**DOI:** 10.1177/23743735241302936

**Published:** 2024-12-09

**Authors:** Noor Fathima Shaik, David Do, Roy Rosin

**Affiliations:** 1 Department of Neurology, Penn Medicine, 21798 University of Pennsylvania, Philadelphia, PA, USA

**Keywords:** patient satisfaction, acts of kindness, burnout

## Abstract

A patient's hospital stay is too often wrapped in fear and worry. Healthcare leaders have emphasized the immense need to improve patient experience and address patients’ individual needs. In addition to helping with the medical aspect of healing, we believe health systems can encourage and empower providers to perform acts of kindness to help elevate the otherwise stressful experience of being hospitalized. We describe an initiative focused on tailoring joyful surprises, like unexpected gifts, to help support both patients and clinicians, aiming to improve patient experience and satisfaction while reducing provider burnout. In sharing the stories of the interactions between the providers and patients, it is clear that not only has this program brought joy to our patients, but that it has also helped reconnect our providers with their sense of meaning and purpose in caring for people and meeting their needs. Thus, we herein describe a patient-centered initiative that enables healthcare providers to provide unique and joyful surprises for their patients in a manner that is readily scalable, cost-effective, accessible, and deeply impactful.

## Key Points

1. **Seek out delight in acts of kindness by** identifying small but individually meaningful ways to brighten a patient's day.
2. **Empower the entire healthcare team** by having any member able to request a gift.
3. **Share the joy and decrease burnout by** reinforcing the humanistic and gratifying aspects of practicing medicine, reconnecting teams to the mission of providing care, and re-energizing staff in the process.

## Introduction

In a recent survey of healthcare executives, clinicians, and clinical leaders, 96% say healthcare can learn about engagement from other consumer-facing industries, especially regarding customer experience and customization to individual needs.^1^ Yet only 18% believed their organizations’ efforts have been very or extremely effective in incorporating patient feedback for improvement, highlighting the large gulf to meet this goal.^2^

With an ever-increasing focus on the consumerization of healthcare, patient-reported outcome measures, and satisfaction scores tied to reimbursement, hospital systems have unsurprisingly tried various techniques to improve patient experience and to incorporate “good surprises,” which have been shown to improve patient satisfaction ratings.^3-8^ But free snacks cannot help re-open blood vessels after a stroke and this blanket approach has been criticized for diverting already limited resources away from patient care and ignoring the individual needs of patients.^6,7^ One suggestion to help handcraft such customized experiences is that “health systems should develop pilot programs that build unique patient-centered delight in order to identify scalable interventions”; as Airbnb did with “superhosts” surprising guests with dinners and transportation, making a trip more meaningful and enjoyable.^9^ Further, social scientists have explored and established how positive surprises—experiences that are unexpected—and those that create emotional resonance, make people feel special or cared for, and drive word of mouth, elevating the likelihood that a service would be recommended to others.^10^

In addition to helping patients, we also wanted to reconnect healthcare professionals with their calling to medicine, especially as this is associated with decreased burnout and increased well-being.^11,12^ Take, for instance, a patient with osteomyelitis who needed an amputation, but unfortunately experienced complications during her hospitalization. The loss of her hair-tie during surgery was the ‘last straw’ that made her distraught. When we surprised her with a simple pack of hair-ties, her tears morphed from ones of frustration to tears of joy and helped the entire team realize how such a small act of kindness can bring meaning and purpose not just to our patients but also to us as clinicians.^13^ Another patient had lost most of his belongings in a fire and soon after was hospitalized from a stroke. His one request before discharge was to wash his clothes, the only set he now owned, and we took them to a laundromat. He sent the team a photo of his reunion with his family wearing that same set of clothes, highlighting how such a simple act can imbue a deeper sense of purpose when it is meaningful and tailored to the patient's needs.

Thus, we sought to personalize delight and encourage acts of kindness for patients in a manner that was easy to do and could be applied across health systems.

## Description of the Intervention

We developed a Web-based form that is freely accessible to employees at the Hospital of the University of Pennsylvania (HUP). Known as PennHOPES within our institution, standing for “Helping Our Patients Smile,” the form is easily accessed via a URL and does not require download or setup. The form asks for the requesting provider's (henceforth termed “requestor”) contact information, the patient's expected length of stay (LOS), the anticipated gift/cost, and the unique challenges faced by the patient. We do not collect any protected health information. The general criteria for a PennHOPES request are listed in Table 1. The average hospital stay in the United States has a mean LOS of 4.6 days and costs thousands of dollars.^14^ Therefore, our initiative was aimed at helping patients with LOS longer than the mean hospitalization, incurring only a nominal additional cost.

**Table 1.** Criteria for Approval of PennHOPES Request.

Criteria for a PennHOPES request
Patient is admitted to the hospital with an expected LOS of at least 5 days
The gift is < $50
The gift is something that can bring the patient joy
Not for drug copays or bills, not for transportation vouchers
Nothing illegal
The gift does not interfere with the patient's medical plan (eg, low sodium diet)

This initiative was supported by a $5000 pilot grant from the Center for Healthcare Transformation and Innovation at Penn Medicine, and Amazon gift cards of $20 or $50 were purchased to allow for quick distribution. Our only condition of the requestors is they share with us the story of how the exchange went when they presented the patient with their gift.

This is analogous to the 3 Wishes Project, wherein clinicians are encouraged and empowered to fulfill 3 wishes for patients at the end-of-life, which often include wishes like favorite foods, blankets, celebrations, etc with a mean cost under $20.^15^ But we sought to broaden the scope to include hospitalized patients at all stages of recovery, across all services.

In August 2023, we introduced PennHOPES to our inpatient neurology team (charge nurses, residents, physician assistants [PAs], and nurse practitioners [NPs]), internal medicine residents/nurses, oncology team (PAs and NPs), and social work (SW)/case management (CM) team. We emailed these groups a note explaining PennHOPES, a link to the form, and general criteria for approval (Table 1), and encouraged them to share this with their colleagues. We selected these initial services since many of our patients with extended stays are admitted to internal medicine services including cardiology, oncology, med-surg (covered by internal medicine residents, nurses, and oncology PAs/NPs), and neurology services including stroke and epilepsy monitoring unit (covered by the inpatient neurology team). Our SW/CM teams span all medical and surgical services across the hospital and often spend time with patients learning about their nonmedical challenges and barriers to care, making PennHOPES an ideal tool in their efforts to help patients. Furthermore, when looking at the top 15 reasons for prolonged hospital stays, at least 12 were mainly medical/neurological conditions that would be seen by the aforementioned selected teams.^16^

Once a request is submitted on the form, it populates a shared document that alerts all members of the PennHOPES team (an interdisciplinary group of residents, SW, and nursing). One of the team members would review the request, ask clarifying questions if needed, and send an email or text message approving or denying the request; on average this approval/denial would usually happen within 2 business hours of the request. Then the gift card was delivered to the requestor, often within 1 business day, either in-person or via e-gift cards. Requestors would get either $20, $40 (two $20 gift cards), or $50 based on the expected cost of their request; they could use this to either directly purchase the gift on Amazon or to purchase elsewhere and reimburse themselves. The person who approved the request would follow-up to ask for the story of when the gift was presented to the patient and how that interaction went.

## Evidence of Impact

Although the program is newly under way and ongoing, since launching it 12 months ago there have been on average 1 to 2 requests weekly averaging $37 each, for a total of 81 requests (79 completed and 2 canceled as patients left earlier than anticipated) placed by 47 unique providers (each placing 1-9 requests), 85 patients helped (as some requestors used one request to help multiple patients), and $3035 granted. By department, around 65% of requests are from medical services (medicine/oncology/neurology), 22% of requests have been from surgical services, and the remaining requests are from an interdisciplinary psychiatry team that caters to patients across both medical and surgical subspecialties. By the role of requestor, 11% are nurses, 53% are SW/CM/consultants, 16% are residents, and 20% are PAs/NPs/attending physicians/medical students. By gift category, activities are the most common request at 43%, clothing/“other” comprise 26%, followed by food/drink at 21% and toys at 10%. Most importantly, we have also been collecting the meaningful stories our team members have shared, and a sample of these is provided in Table 2 and some stories were also shared in articles.^17,18^ Many providers shared how being able to give these small gifts to their patients improved their own days, brought them joy, and made them feel empowered to do even more for our patients; qualities which were described by participants of other patient-centered projects and are helpful for combatting burnout.^19,20^ Finally, articles about PennHOPES sparked so much enthusiasm from readers that several people reached out to donate to the program.^17,18^

**Table 2.** Examples of Stories Shared by Team Members After Giving Patients Their Gifts.

Gift and cost	Patient circumstances	Story
Slippers and socks; $20	Patient with stroke, no family, birthday coming up	As a senior resident, interacting with patients can be limited. Patient interactions are one of the most gratifying parts of being a doctor! I stopped by a patient's room to figure out who her emergency contacts were—her son died and her sister was not answering her phone. I realized her birthday was the next day. With PennHOPES, I was able to buy my patient a pair of slippers and socks. She was very reluctant to go to rehab, citing her inability to have her normal clothes at the rehab. She loved the gift and gave our team a hug. It brought me more joy than I anticipated and made my otherwise difficult day much better.
Specialty coffee (patient's favorite drink); $10	The patient has been hospitalized throughout most of the summer. Multiple rapid response and intensive care unit readmissions. No discharge date in sight.	Her nurses surprised her with her favorite specialty coffee. She just got out of the operating room when they gave it to her, and it made her day! She gave them a hug and took a photo with them holding up her coffee, beaming with happiness despite her recent surgery.^20^
Keychain brain kit; $20	Challenging social situation, prolonged intensive care unit admission, family is working and unable to visit often.	The patient was so surprised and thankful when we brought him the keychain/braid kit. He said, “Oh My Goodness, this is exactly what I used to do with my grandmother” and became slightly tearful. The staff are reporting he's been using them a lot and has really helped him find something to do while admitted as he has frequent admissions.
New set of clothing, $50	Prolonged hospitalization for substance use, unstable housing, lack of support.	My patient has been struggling with substance use and unstable housing since coming to Philadelphia earlier this year and he does not have much support in this area. His clothing was damaged during admission, and he was very grateful for the clothing I was able to purchase for him with the PennHOPES gift card. He was very appreciative that the hospital is able to help with the various needs that he has.
Stuffed animal; $20	A young patient with epilepsy was having multiple seizures daily and had been hospitalized for most of the month. Minimally verbal and very scared from all the seizures she was having.	Her mother said she has a collection of stuffed animals at home, so a small stuffed goat was presented to her. It made her so happy she cried; her mom said, “These are happy tears because of the kindness.” I am now crying because I am so happy. I love that this initiative is inspiring us to think creatively for how to reach out to our patients.
Spanish-English Visual Dictionary; $35	The patient had a difficult time communicating his needs as he would often have to wait for a provider to use a translator, which compounded his medical challenges.	He's been so happy getting his visual dictionary and has been showing it to everyone. Thank you for helping to run this program—it is such a nice idea and makes patients really happy. And we have been sharing this among our group of medical students to spread the word about this great way to help patients!

## Improvements

The implementation could be improved by using electronic gift cards for rapid delivery and more promotion between different services, perhaps by e-mailing all charge nurses. While we intentionally did not use surveys to limit the burden for our requestors and only asked how the gifting interaction went, others may wish to use more quantitative surveys. We finally hope to identify which services are “super-users” and which gifts are frequently requested to see if there should be a ready inventory of oft-requested items in the future.

## Conclusion

Health systems can easily adapt this program by starting with a form, email, or message-based request platform. This request should be shared with a group who will screen it based on criteria similar to Table 1. Prepurchased gift cards can be distributed quickly and easily to the requestors.
